# Give it a REST!

**DOI:** 10.7554/eLife.12615

**Published:** 2016-01-08

**Authors:** Steven M Pollard, Maria Angeles Marques-Torrejon

**Affiliations:** MRC Centre for Regenerative Medicine, University of Edinburgh, Edinburgh, United Kingdomsteven.pollard@ed.ac.uk; MRC Centre for Regenerative Medicine, University of Edinburgh, Edinburgh, United Kingdom

**Keywords:** transcription factors, REST complex, repression, genomic instability, neurogenesis, knockout animals, Mouse

## Abstract

The REST protein helps to prevent the premature activation of genes that are only expressed in mature neurons, and is now found to protect the genome of neural progenitor cells.

**Related research article** Nechiporuk T, McGann J, Mullendorff K, Hsieh J, Wurst W, Floss T, Mandel G. 2016. The REST remodeling complex protects genomic integrity during embryonic neurogenesis. *eLife* **5**:e09584. doi: 10.7554/eLife.09584

**Image** Brain section showing a tumour caused by the loss of the proteins REST and p53 from apical progenitor cells.

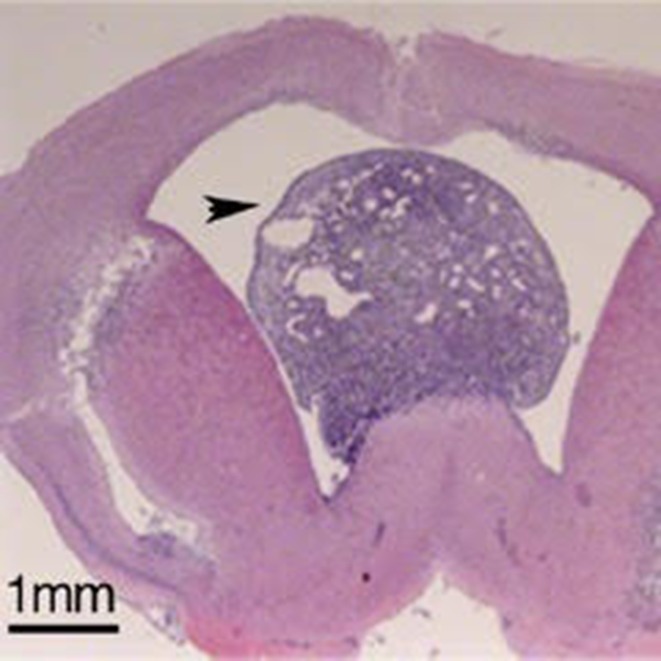

To construct the mammalian brain, the right neurons must be produced in the right place at the right time. For example, the outer layer of the brain, the cortex, consists of six layers of neurons and is built up one layer at a time. This is achieved by controlling how cells called apical progenitors become basal progenitors, which then specialize into new neurons (Gotz et al., 2002; Figure 1). If the apical progenitors become basal progenitors too soon, several developmental brain abnormalities can result, the brain may be too small (a condition known as microcephaly), or the layering of the cortex may be disrupted. Now, in eLife, Gail Mandel of the Oregon Health and Science University and co-workers – including Tamilla Neichiporuk as first author – report that a protein called REST has an unexpected role in protecting the genome of these progenitor cells (Nechiporuk et al., 2016).

**Figure 1.**
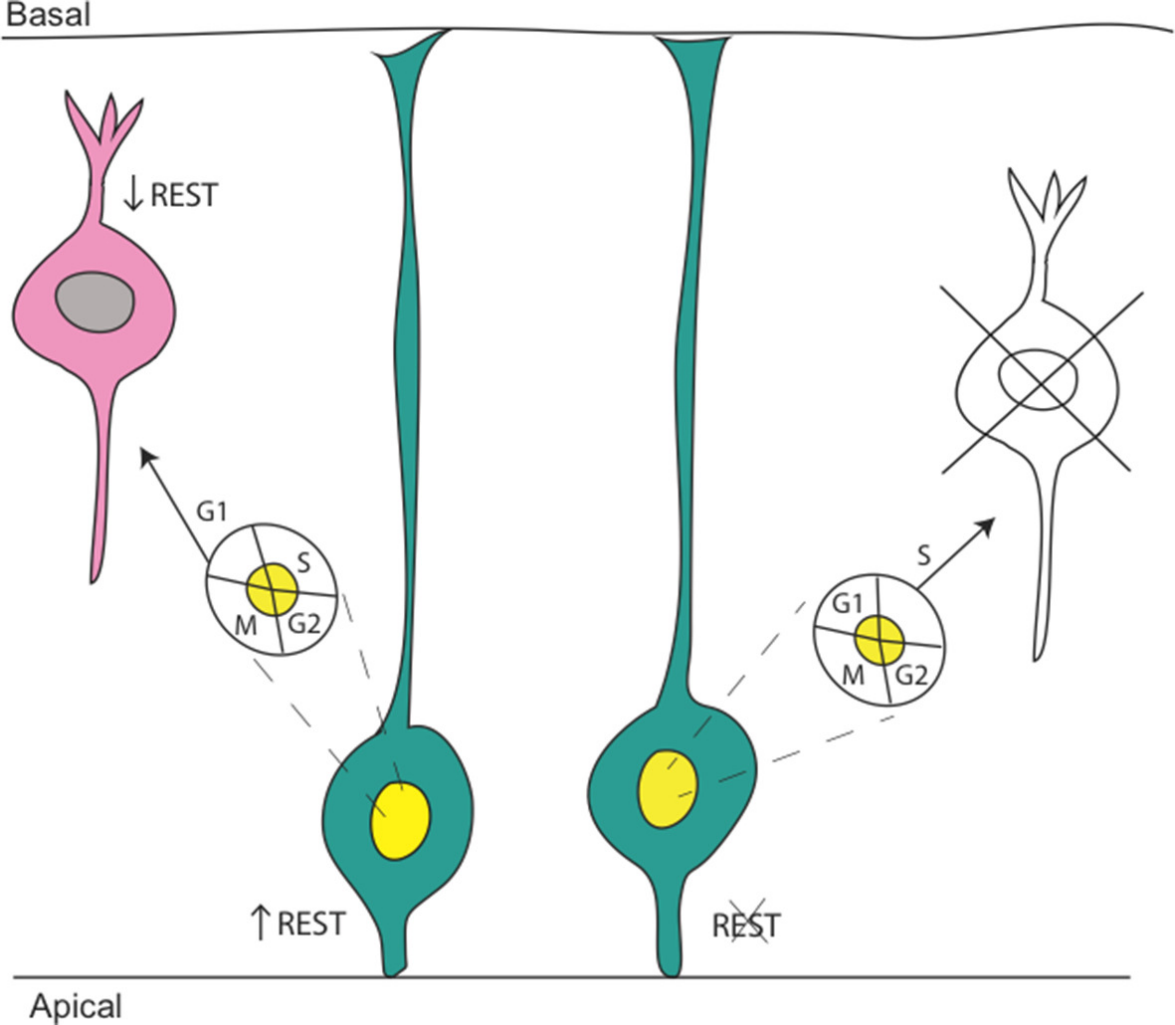
REST regulation in embryonic neurogenesis. The REST protein is expressed in apical progenitors (green cells) and represses the expression of genes that are necessary for neurons (pink cell) to develop. Left: REST maintains genome stability, protecting the DNA of the apical progenitor cell during S phase (the phases of the cell cycle are shown in the yellow and white oval). The cells then reduce the production of REST precisely at the apical progenitor cell cycle exit (during G1 phase). This ensures that new neurons develop properly. Right: In the absence of REST, the DNA of the apical progenitor cell becomes damaged during S phase, preventing the subsequent production of basal progenitors and neurons.

The identity of a cell is determined to a large extent by which of its genes are transcribed. Therefore, when a new neuron first develops from a progenitor cell, a process of 'transcriptional resetting' must occur so that the genes that need to be expressed in mature neurons can be activated. The REST protein, first identified in 1995, is expressed in all cells except for mature neurons, and so researchers immediately suspected that it was involved in repressing neuronal genes (Schoenherr and Anderson, 1995; Chen et al., 1998; Bruce et al., 2004).

Support for this idea came from experiments that showed that REST binds to and represses genetic elements that are associated with many genes that are specific to mature neurons. As part of this repression, REST recruits a series of repressor complexes that alter how the gene is packed into a structure called chromatin. However, a previous study involving knockout mice who could not produce the REST protein failed to identify any significant abnormalities in the developing or adult brain (Gao et al., 2011; Aoki et al., 2012: Yang et al, 2012). The role of REST has therefore remained uncertain.

Nechiporuk et al. – who are based in the US and Germany – have now used a technique called conditional genetic ablation to explore the role of REST in neural progenitors. This revealed an unexpected requirement for REST in protecting the genome of the apical progenitors. Loss of REST induces DNA damage during the S phase of the cell cycle: this is the phase during which DNA is replicated. A consequence of this damage is the acquisition of chromosomal abnormalities in the apical progenitors. This triggers cells to commit suicide – following the orders of a protein called p53 – and the result is microcephaly. Nechiporuk et al. also show that the combined loss of REST and p53 results in the formation of a highly aggressive brain tumour called a glioblastoma. REST therefore performs a dual role during brain development: it protects the progenitor cells from genetic catastrophe, and it silences neuronal gene expression until the time is right.

These findings came as a surprise, given the results of the previous REST knockout studies. However, in a series of elegant experiments Nechiporuk et al. showed that these earlier mouse models did not fully delete the REST coding sequences: the knockout mice still produced a C-terminal peptide that was able to recruit the repressor complexes that helped to silence certain genes. This is a valuable lesson for all researchers – knocking out a gene does not always result in a complete loss of function.

Why does the premature removal of the repressor complexes recruited by REST inflict widespread genomic damage? One possibility proposed by Nechiporuk et al. is that the associated loss of chromatin repression might lead to a subset of neuronal genes being incorrectly transcribed. Thus, REST seems to provide a ‘license’ for progenitors to transform into their final neuronal form by guarding the genome and preventing the premature transcription of genes specific to mature neurons. These new findings address a question that has received little attention to date – how are genome maintenance and transcriptional control coordinated as new neurons develop from progenitor cells?

As with all interesting discoveries, many new questions arise: how is REST protecting neuronal gene integrity during S-phase? How are the processes of cell cycle exit, chromatin repression, and the DNA replication timing coordinated during the birth of new neurons? New insights into how REST orchestrates gene regulation during the construction of the nervous system will clearly enhance our understanding of diseases such as microcephaly and brain cancer. It seems that for neural progenitors, a little REST is what it takes to ensure you reach your full potential!
